# AI and Blockchain as New Triggers in the Education Arena

**DOI:** 10.3390/ejihpe12040032

**Published:** 2022-04-08

**Authors:** Maria José Sousa, Francesca Dal Mas, Sónia P. Gonçalves, Davide Calandra

**Affiliations:** 1 Department of Political Science and Public Policies, ISCTE-Instituto Universitário de Lisboa, 1649-026 Lisboa, Portugal; 2 Department of Management, Ca’ Foscari University, Cannaregio, 873, 30100 Venice, Italy; francesca.dalmas@unive.it; 3 Instituto Superior de Ciências Sociais e Políticas, Universidade de Lisboa, Centro de Administração e Políticas Públicas, 1300-663 Lisboa, Portugal; sonia-goncalves@campus.ul.pt; 4 Department of Management, Università degli Studi di Torino, Corso Unione Sovietica 218 bis, 10134 Torino, Italy; davide.calandra@unito.it

Several scholars have examined the potential use of AI and Blockchain in education, primarily focusing on the contributions of such technologies with a goal to improve learning possibilities and outcomes for students. Such technologies may be utilized to ensure that all students have equal access to education, including people with disabilities, refugees, and those living in isolated communities or rural areas. AI-empowered applications such as holograms and robotics may enable children with special needs to attend school from home or from hospital, as well as ensure learning continuity in emergencies or crises, such as during the recent COVID pandemic. Moreover, teachers and lecturers can utilize such new systems to monitor asynchronous discussion groups, boosting the involvement of the participants through Intelligent Tutoring Systems, making group discussions more engaging even at a distance, coupled with e-learning. In larger cohorts, AI can support the fast grading of tests and homework and can provide tools to prevent cheating.

While several universities and institutions worldwide are testing new AI and Blockchain applications to increase students’ learning outcomes and educational experience, the use of such technologies in the educational field is still in its infancy, and much still needs to be explored, tested, and shared.

Therefore, the goal of this Special Issue (SI), “AI and Blockchain as New Triggers in the Education Arena”, was to foster dialogue in the promising field of AI and Blockchain in the educational sector as new triggers in the development of new approaches to academic management, learning contexts, and AI- and Blockchain-based technology applications and tools devoted to education.

The SI includes three articles (Table 1) that discuss the application level of new technologies such as AI and Blockchain in the education sector. In terms of contributions collected concerning AI, we indirectly point to the need for more studies and insights in the future. Although this technology is proving to be of high value at the level of practical applications in several sectors such as health care, accounting, finance, and the service sector, in the educational sector, further studies and practical insights are needed to legitimise the scientific development of AI in this area. Finally, as in the case of as-yet unexplored research areas, it may be helpful to apply systematic methodologies of analysis focusing on both the academic and practitioner spheres. This would make it possible to analyze practitioners’ challenges and allow case researchers to scientifically validate and modify significant theories.

In terms of contributions collected regarding the Blockchain theme, the SI uncovered some interesting findings (Table 1).

The first contribution is a bibliometric analysis by Reis-Marques and colleagues [1] conducted on 61 research articles demonstrating a nascent and promising field of co-knowledge. The implications of the most cited articles are interesting. For example, they demonstrate how Blockchain can be easily used to protect digital certificates from fraud. The research paper is a collaboration between academia and practitioners, particularly the Taiwan Ministry of Education, and demonstrates proactive cooperation to increase the security of school certificates. Additionally, they show how Blockchain can be used to certify content and validate skills between academia and the world of work. Alongside this, applications for lifelong learning and new platforms to certify training processes are also emerging. The results proposed by the authors are also interesting, as they suggest the creation of an educational consortium based on Blockchain technology. The model allows for the centralized verification of students’ academic certificates and verified and secure study information to be shared with the world of work.

The second contribution published by Raimundo and colleagues [2] systematizes the knowledge flow of Blockchain in higher education by uncovering 37 research articles. Compared to the previous analysis, the authors add ways to share and protect educational data. In particular, the authors find that Blockchain can play a role both internally (i.e., among students themselves) and externally (i.e., with various stakeholders such as companies). Finally, their contribution focuses on the critical potential that Blockchain may have for the technological improvement and security of student career data management.

Finally, the third contribution by Castro and Au-Yong-Oliveira [3] starts by observing an increase in student mobility. This potentially poses problems in the monitoring of higher education certificates and diplomas. The authors take note of this research problem and investigate, using thematic review tools, the developments of a possible link between Blockchain and higher education diplomas. Their results also confirm that technology can positively impact refugees who could benefit from a protected educational history within Blockchain nodes. Thus, technology can increase students’ quality of life worldwide by consolidating mobility processes and speeding up the recognition of academic qualifications.

The goal of this Special Issue was to create a discussion in the field of Artificial Intelligence and Blockchain in education regarding new approaches to academic management, learning contexts, and AI and Blockchain technology application to education. In this sense, this Special Issue provides a global forum for the investigation and reporting of diverse issues that affect the application of AI and Blockchain to education: innovations in the learning processes, new avenues for education managers, and new learning contexts. The main outputs of the SI are original research contributions in the form of research papers and demonstrations of original scientific results—specifically, studies in education regarding the use of Blockchain technologies, new educational management systems, new tools, and the best practices of AI and Blockchain learning. Finally, this SI gained the attention of scholars in several scientific fields and will hopefully become a forum for the discussion and analysis of AI and Blockchain in education.

## Figures and Tables

**Table 1:** Special Issue articles: authors, titles, and keywords.

Author	Title	Keywords
Reis-Marques and colleagues (2021) [1]	Applications of Blockchain Technology to Higher Education Arena: A Bibliometric Analysis	Blockchain technology, bibliometric studies, disrupt higher education, digital transformation
Raimundo and colleagues 2021 [2]	Blockchain System in the Higher Education	Blockchain, education, higher education
Castro and Au-Yong-Oliveira (2021) [3]	Blockchain and Higher Education Diplomas	Blockchain, higher education, diplomas, certificates, fraud, international students, radical innovation, refugees
